# *De novo* sequencing and transcriptome analysis of the desert shrub, *Ammopiptanthus mongolicus*, during cold acclimation using Illumina/Solexa

**DOI:** 10.1186/1471-2164-14-488

**Published:** 2013-07-18

**Authors:** Tao Pang, Chu-Yu Ye, Xinli Xia, Weilun Yin

**Affiliations:** 1 College of Biological Science and Biotechnology, National Engineering Laboratory for Tree Breeding, Key Laboratory for Silviculture and Conservation, Beijing Forestry University, Beijing 100083, China

**Keywords:** *Ammopiptanthus mongolicus*, Cold acclimation, Transcriptome, Illumina/Solexa

## Abstract

**Background:**

*Ammopiptanthus mongolicus* (Maxim. ex Kom.) Cheng f., an evergreen broadleaf legume shrub, is distributed in Mid-Asia where the temperature can be as low as −30°C during the winter. Although *A. mongolicus* is an ideal model to study the plant response to cold stress, insufficient genomic resources for this species are available in public databases. To identify genes involved in cold acclimation (a phenomenon experienced by plants after low temperature stress), a high-throughput sequencing technology was applied.

**Results:**

We sequenced cold-treated and control (untreated) samples of *A. mongolicus*, and obtained 65,075,656 and 67,287,120 high quality reads, respectively. After *de novo* assembly and quantitative assessment, 82795 all-unigenes were finally generated with an average length of 816 bp. We then obtained functional annotations by aligning all-unigenes with public protein databases including NR, SwissProt, KEGG and COG. Differentially expressed genes (DEGs) were investigated using the RPKM method. Overall, 9309 up-regulated genes and 23419 down-regulated genes were identified. To increase our understanding of these DEGs, we performed GO enrichment and metabolic pathway enrichment analyses. Based on these results, a series of candidate genes involved in cold responsive pathways were selected and discussed. Moreover, we analyzed transcription factors, and found 720 of them are differentially expressed. Finally, 20 of the candidate genes that were up-regulated and known to be associated with cold stress were examined using qRT-PCR.

**Conclusions:**

In this study, we identified a large set of cDNA unigenes from *A. mongolicus*. This is the first transcriptome sequencing of this non-model species under cold-acclimation using Illumina/Solexa, a next-generation sequencing technology. We sequenced cold-treated and control (untreated) samples of *A. mongolicus* and obtained large numbers of unigenes annotated to public databases. Studies of differentially expressed genes involved in cold-related metabolic pathways and transcription factors facilitate the discovery of cold-resistance genes.

## Background

*Ammopiptanthus mongolicus* (Maxim. ex Kom.) Cheng f. is an endangered angiosperm genus (Leguminosae) mainly distributed in the deserts of eastern central Asia [1]. As the only evergreen broad-leaved shrub in this area, it is particularly important for the ecological-environmental stability of native habitats [2]. Moreover, the increasing desertification in central Asia is gradually becoming more serious in recent years [3]. Due to the important role that *A. mongolicus* plays in fixing moving sands and delaying further desertification, the protection and research on *A. mongolicus* are becoming particularly important and necessary [4]. Former studies on *A. mongolicus* have shown its capacity of resisting high solar radiation, heat, cold, and drought stresses [5,6]. Some genes involved in drought, cold, heat, and salinity tolerance have been identified, such as *AmCBL1*[7], *AmLEA*[8]*AmVP1*[9], *AmNHX2*[10] and *AmCIP*[11]. However, these studies on *A. mongolicus* are still limited and genomic resources of *A. mongolicus* (749 EST and 155 nucleotide sequences in GeneBank prior to 11 January 2013) are also scarce. The ability to survive at −30°C or lower temperatures makes *A. mongolicus* an ideal model for studying mechanisms of cold tolerance in plants. Therefore deeper understanding on genes expression profile of *A. mongolicus* under cold stress would be significative and imperative.

Cold acclimation; *i*.*e*., enhancement of the freezing tolerance of plants after low temperature stress, has been observed in many plants, including *Arabidopsis*[12], *Oryza sativa*[13], *Triticum aestivum*[14], and *A. mongolicus*[15]**.** During this process, plants alter the expression of certain genes as well as the biosynthesis of amino acids and soluble sugars. To elucidate the mechanism underlying cold acclimation, it is important to determine how plants alter gene expression in response to this biological process [16].

Compared with the traditional Sanger method, which is expensive and time consuming, next-generation sequencing (NGS) technologies or massively parallel sequencing technologies (*e*.*g*. Illumina/Solexa-based RNA-Seq technology) are much simpler and more cost-effective [17]. Furthermore, these high-throughput RNA sequencing (RNA-Seq) technologies have other advantages, such as accuracy and sensitivity for both low- and high-level gene expression [18], and facilitate rapid identification and analysis of the vast majority of transcriptomes [19]. Three main commercially available next-generation sequencing technologies are extant; namely, ABI/SOLiD, 454/Roche and Illumina/Solexa [20]. Illumina/Solexa has been successfully applied to transcriptome sequencing of many plant species, including *Populus euphratica*[21], *Aegilops variabilis*[22] , *Brassica napus*[23], *Zea mays*[24], *Arachis hypogaea*[25], and *Picrorhiza kurrooa*[26].

Here, we describe the first transcriptome sequencing of *A. mongolicus* under cold-acclimation conditions using a next-generation sequencing technology, Illumina/Solexa. We sequenced two cDNA libraries (cold-treated and control samples) of living *A. mongolicus* tissues, and got an unprecedented amount of data. All sequences were deposited in Short Read Archive (SRA) division of the GenBank repository (accession no. SRA064010). Genes identified in this study expanded the available EST resources of *A. mongolicus*. Moreover, the analyses on differentially expressed genes under cold stress also furthers our understanding of the cold response mechanism of *A. mongolicus*, and the cold-related genes would also contribute to provide a method of developing cold-tolerant plants through genetic manipulation.

## Results

### Genome-size estimates

We determined the genome size of *A. mongolicus* by flow cytometry using cotyledons nuclei with maize (≈2500 Mb [27]) as an internal standard. The result showed that the genome size of *A. mongolicus* (2n = 18) was approximately 819.56 ± 7.61 Mb (Table 1), which was similar to that of another legume, the chick pea (*Cicer arietinuum*, ≈738 Mb) [28]. This is the first report of the genome size of *A. mongolicus* as far as we know. It is helpful to determine the sequencing depth.

**Table 1:** **Flow cytometry determination of the nuclear genome sizes of *A. mongolicus***

***A. mongolicus *peak**	**Reference peak**^**1**^	**Peak ratio (Ammopiptanthus /reference)**	***A. mongolicus *genome size (1C, Mb, ±standad deviation)**
15.53	47.07	0.329934141	824.8353516
16.48	50.06	0.329204954	823.0123851
15.62	48.16	0.324335548	810.8388704
			819.56 ± 7.61

^1^Nuclei from maize young leaves serve as a size standard, which has a haploid genome size of 2500 Mb.

### Illumina sequencing and reads assembly

The two libraries (cold-treated and control samples), were sequenced respectively using Illumina HiSeq™ 2000. The total clean nucleotides generated from each sample exceeded 5.8 Gb, that is, an unprecedented depth of seven times (7-fold or 7× coverage) as much as the genome size (819.56 ± 7.61 Mb). We obtained approximately 71 million raw reads for the cold-treated sample (CT) and 73 million for the control sample (CK). We discarded low-quality reads, which contained adapters and unknown or low-quality bases, according to our bioinformatics analysis. A total of 65 million and 67 million clean reads were obtained from CT and CK samples, respectively. Of these clean reads, the total length was 11.9 × 10^9^ nt and the Q20 percentage (percentage of sequences with sequencing error rate lower than 1%) was over 97% for both samples. All clean reads were also deposited in the National Center for Biotechnology Information (NCBI) and can be accessed in the Short Read Archive (SRA) under the accession number SRA064010.

**Figure 1:**
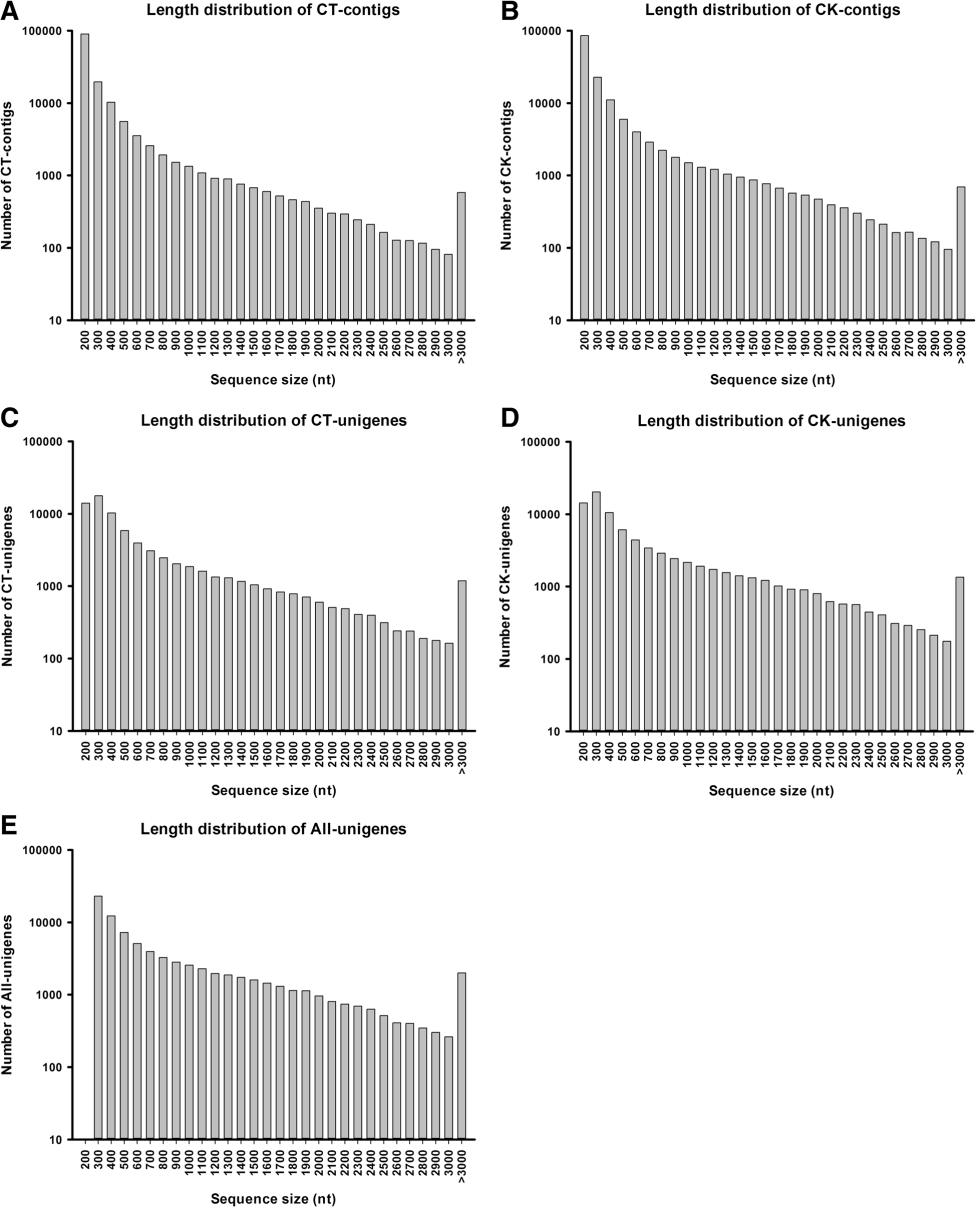
**Length distribution of the contigs and unigenes.** The length distribution of contigs of CT sample **(A)**, contigs of CK sample **(B)**, unigenes of CT sample **(C)**, unigenes of CK sample **(D)**, and the all-unigenes **(E)**.

Transcriptome *de novo* assembly was performed using Trinity, a short reads assembling program [29], which generated 145,000 (CT) and 148,797 contigs (CK) (Table 2). In both samples, the average contig size exceeded 300 nt, with the N50 of 500 nt. The contigs of each sample were then connected into unigenes, generating 76000 (CT) and 84583 unigenes (CK), respectively. After long-sequence clustering between both samples, 82795 all-unigenes were obtained. The sequencing coverage ranged from 1- to 66245-fold (average, 45-fold). The total length was 67,554,337 nt, with a mean length of 816 nt and an N50 of 1343 nt (i.e., 50% of the assembled bases were incorporated into unigenes of 1343 nt or longer). Each all-unigene was longer than 200 nt, and 35350 (42.70%) unigenes were 200 to 400 nt. Also, 8066 (9.74%) unigenes were longer than 2000 nt. The size distribution of the contigs and unigenes is shown in Figure 1.

**Table 2:** Overview of the sequencing and assembly

	**Cold-treated**	**Control**	**Total**
Total Raw Reads	71,441,910	73,279,028	
Total Clean Reads	65,075,656	67,287,120	
Total Clean Nucleotides (nt)	5,856,809,040	6,055,840,800	
Q20 percentage	97.39%	97.60%	
N percentage	0.00%	0.00%	
GC percentage	45.87%	45.40%	
**Contig**			
Total Number	145,000	148,797	
Total Length(nt)	45,723,903	51,308,749	
Mean Length(nt)	315	345	
N50	521	619	
**Unigene**			
Total Number	76,000	84,583	82,795
Total Length(nt)	48,956,203	57,108,594	67,554,337
Mean Length(nt)	644	675	816
N50	1122	1191	1343
Total Consensus Sequences	76,000	84,583	82,795
Distinct Clusters	1,831	2,043	40,988
Distinct Singletons	74,169	82,540	41,807

### Functional annotation and classification

**Figure 2:**
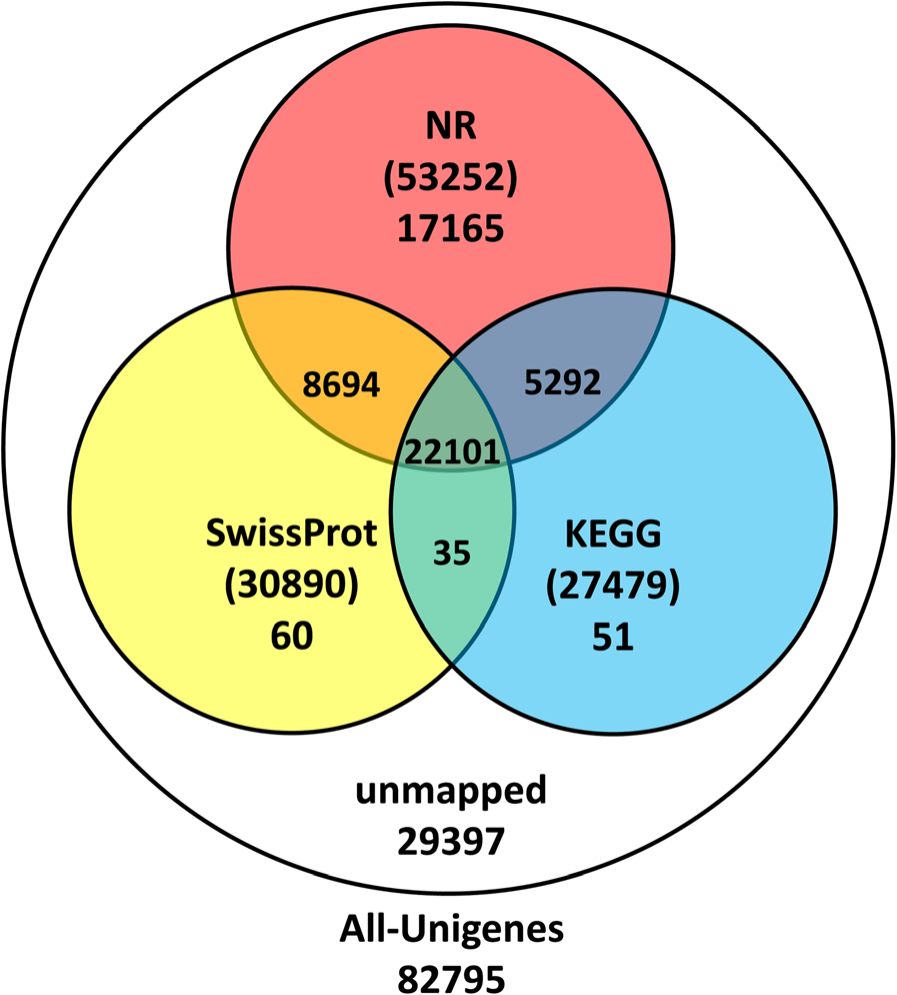
Number of unigenes blasted to NR, Swiss-Prot, KEGG and COG (E < 0.00001).

We next performed BLAST (E-value < 0.00001) analysis of the 82795 all-unigenes against protein databases, following a priority order of Nr (non-redundant protein sequences in NCBI), Swiss-Prot, KEGG (Kyoto Encyclopedia of Genes and Genomes database), and COG. There were 53398 (64.5%) unigenes with homologous sequences in at least one of the above databases. Among them, 53252 (64.3%), 30890 (37.3%), and 27479 (33.2%) unigenes were found in NR, SwissProt, and KEGG, respectively. A total of 22101 (26.7%) unigenes were found in all three databases, while 29397 (35.5%) unigenes were not identified (Figure 2).

We analyzed the 100 most-abundant transcripts of each sample and found some differences in functional annotations. Compared with control sample, the most-abundant transcripts in the cold-treated sample were dehydrin, the LEA protein precursor, SRC1 protein, defensin D1, early light inducible protein, SOS2-like protein kinase, Ethylene-responsive transcription factor, and sucrose synthase. The 100 most-abundant transcripts are listed in Additional file 1.

We also identified a total of 1322 unigenes genes expressed only in the cold-treated sample. Functional annotations showed that some of them were closely related to cold stress, including calcium-transporting ATPase, serine/threonine-protein kinase, CBL-interacting protein kinase, late embryogenesis abundant protein, and dehydrin. Unigenes expressed only in the cold-treated sample are listed in Additional file 2.

**Figure 3:**
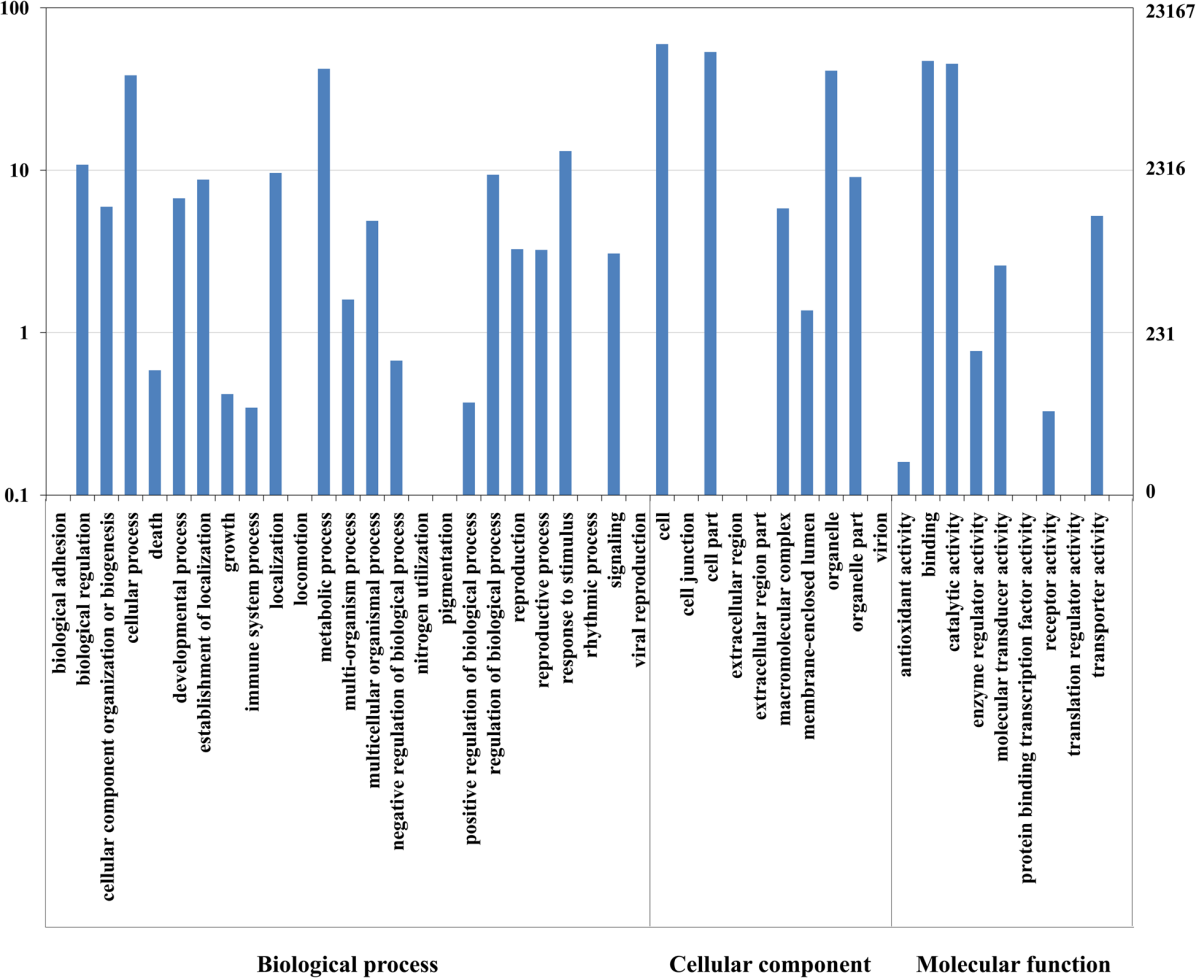
**Histogram presentation of Gene Ontology classification.** The results are summarized in three main categories: biological process, cellular component, and molecular function. The y-axis on the right side indicates the percent of genes in a category, and the y-axis on the left side means the number of genes.

Based on Nr annotations, we used the Gene Ontology (GO) classification system to classify the possible functions of the unigenes. A total of 23167 (28.0%) unigenes were successfully assigned to at least one GO term annotation (Figure 3). The unigenes were then classified into three main categories: biological processes, cellular components, and molecular function. The category of biological processes consisted of 944 GO terms, which were assigned to 14719 (17.8%) unigenes. The cellular components category consisted of 196 GO terms, which were assigned to 14095 (17.0%) unigenes. The category of molecular functions consisted of 577 GO terms, which were assigned to 18775 (22.7%) unigenes. For biological process, the top five largest categories were: “metabolic process” (9731), “cellular process” (8907), “response to stimulus” (3037), “biological regulation” (2502), and “localization” (2226). For cellular components, the top three largest categories were: “cell” (13821), “cell part” (12354), and “organelle” (9472). For molecular function, the top three largest categories were: “binding” (10876), “catalytic activity” (10452), and “transporter activity” (1215).

**Figure 4:**
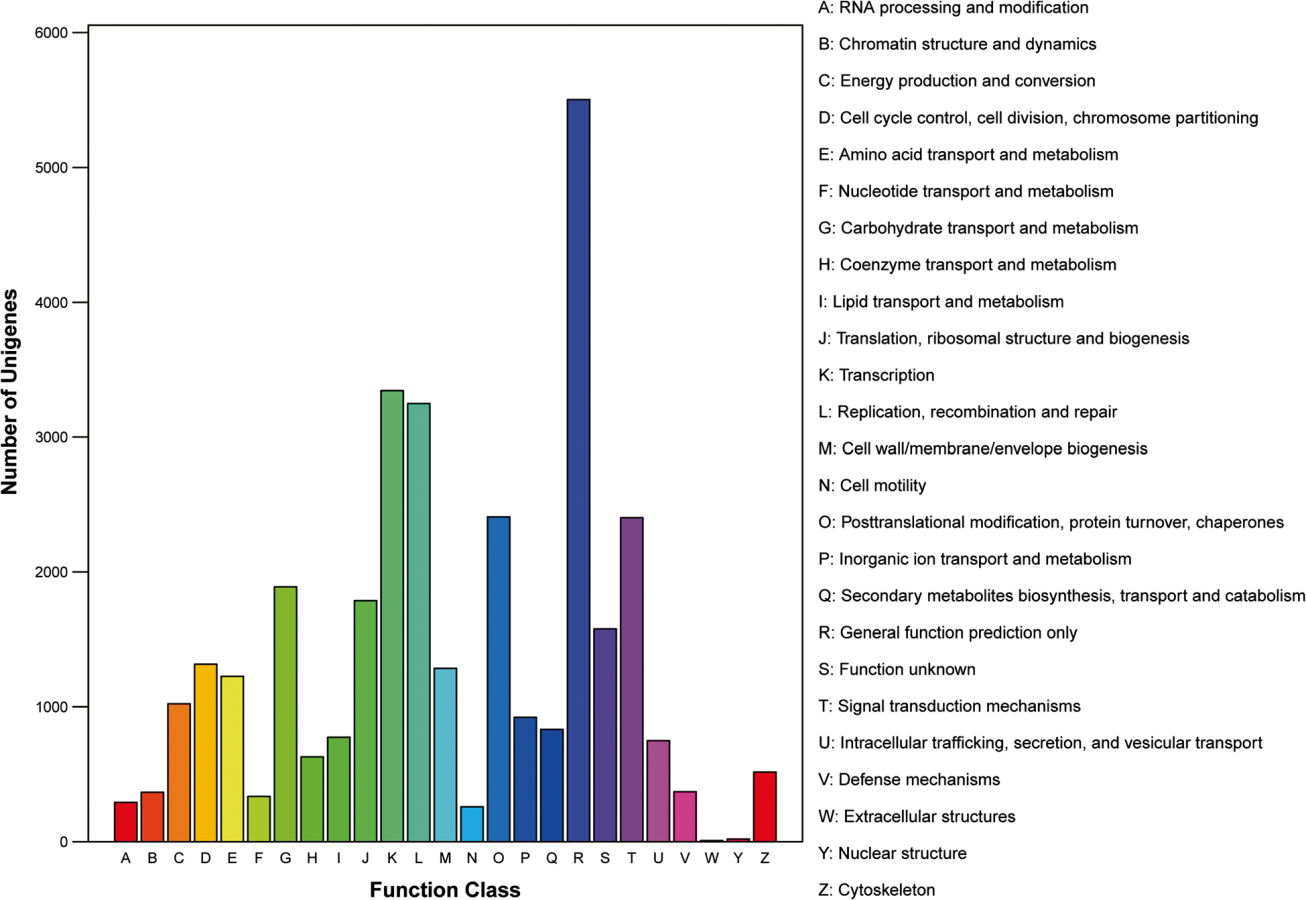
**COG classificationf.** A total of 17327 unigenes were assigned to 25 classifications. The capital letters in x-axis indicates the COG categories as listed on the right of the histogram, and the y-axis indicates the number of unigenes.

To assess the integrality of our transcriptome library and effectiveness of the annotation process, we aligned the all-unigenes to the COG database and 17327 (20.9%) were identified. By classifying the possible functions of these unigenes, they were grouped into 25 functional categories (Figure 4). The largest category was “General function prediction only” (5504 of 17327 unigenes, about 31.8%), followed by “Transcription” (3344 unigenes, about 19.3%), “Replication, recombination and repair” (3248, 18.7%), “Post-translational modification, protein turnover, chaperones” (2408, 13.9%), and “Signal transduction mechanisms” (2402, 13.9%). The categories of “Extracellular structures” (8, 0.046%), “Nuclear structure” (20, 0.12%) and “Cell motility” (257, 1.48%) had the fewest responding genes. Also, 1576 (9.10%) unigenes were annotated as “Function unknown”.

We performed BLAST analysis of the 82795 all-unigenes against the KEGG database to further analyze gene products during metabolic processes and determine their functions in cellular processes. A total of 27479 (33.2%) unigenes matched 255,974 members, which were involved in 125 KEGG pathways. Of the 27479 unigenes, 5905 (21.49%) were related to metabolic pathways, 2821 (10.27%) were related to the biosynthesis of secondary metabolites, 1667 (6.07%) to plant hormone signal transduction, 1060 (3.86%) to RNA transport, and 975 (3.55%) to the spliceosome.

### Protein-coding region prediction

**Figure 5:**
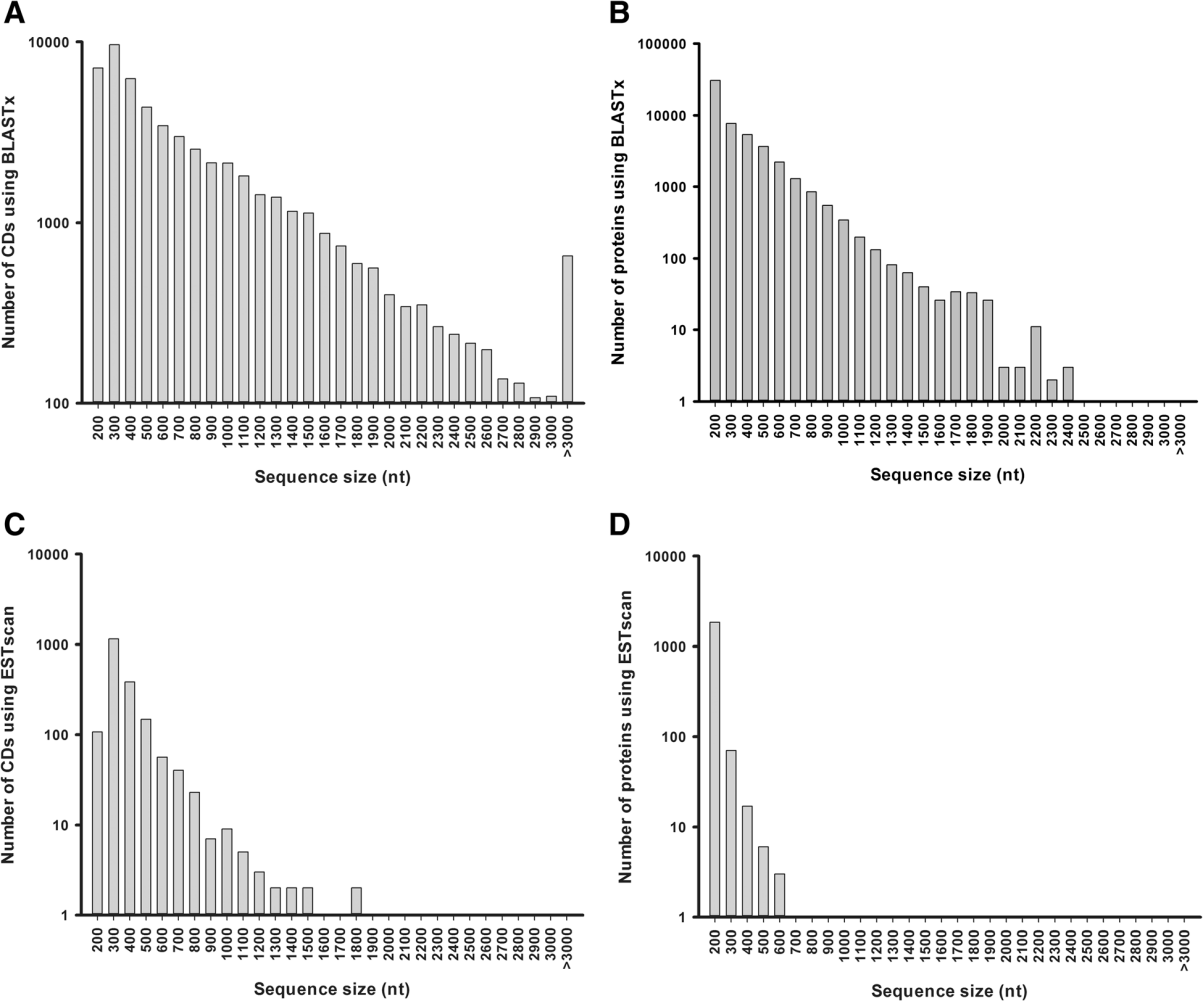
**Length distribution of the protein coding region prediction (CDS). A.** The length distribution of CDs using BLASTx. **B.** The length distribution of proteins using BLASTx. **C.** The length distribution of CDs using ESTscan. **D.** The length distribution of proteins using ESTscan.

After searching all-unigene sequences against protein databases using BLASTx (E-value < 0.00001) in the order mentioned in the functional annotation and classification section, we extracted 53485 coding sequences (CDS) from unigene sequences and translated them into peptide sequences. For unigenes with no BLAST hits, we used ESTScan to predict the 1936 CDS and translated them into peptide sequences. The distribution of the CDS is shown in Figure 5.

### Differential expression analysis

**Figure 6:**
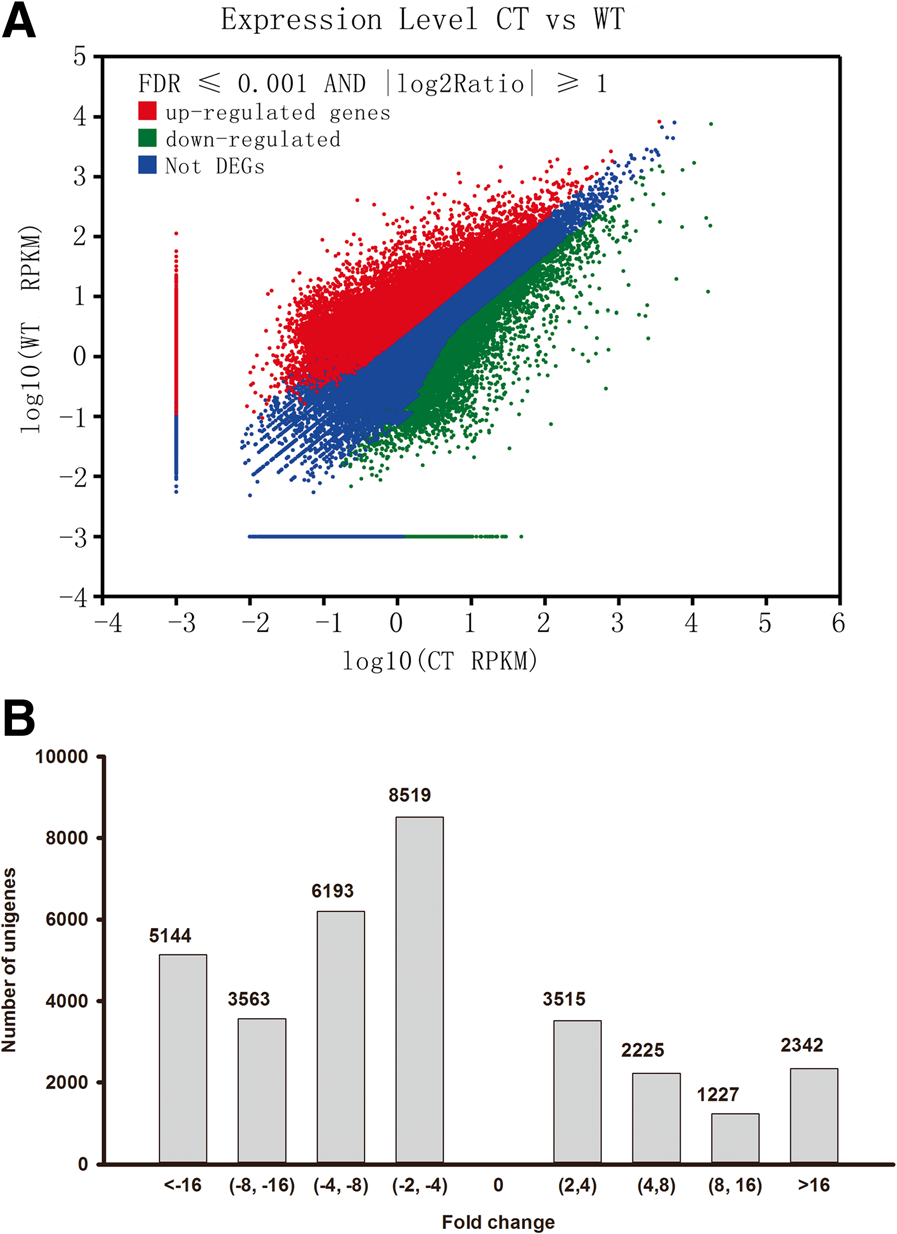
**Distribution of transcript changes in cold-stress sample compared with control sample. A.** The expression levels of all-unigenes **B.** The distribution of differentially expressed genes.

To identify genes with different expression levels, we used the RPKM method (Reads Per kb per Million reads) to calculate the expression levels of the unigenes. The result showed that 9309 genes were up-regulated and 23419 genes were down-regulated with FDR ≤ 0.001 and ratios larger than 2. The distribution of transcript changes is shown in Figure 6.

We then mapped all differentially expressed genes to each term of the Gene Ontology database (http://www.geneontology.org/, release data: Aug 1st, 2012) and calculated the gene numbers from each GO term. Using a hypergeometric test, we identified significantly enriched GO terms in DEGs compared to the genomic background. GO terms with a corrected *p* value ≤ 0.05 were defined as significantly enriched in DEGs. The GO enrichment analysis results are shown in Additional file 3.

We also performed metabolic pathways enrichment analysis, and identified the primary biochemical pathways and signal transduction pathways in which DEGs involved. A total of 3868 up-regulated unigenes and 11691 down-regulated unigenes were identified to be involved in cold stress, on which all following analyses and discussions of metabolic pathway were based. These genes were related to 44 metabolic pathways, showing significantly changed under cold stress (*p* ≤ 0.05) including genes involved in carbohydrate, amino acid metabolism, energy, lipid, confactors and vitamins, terpenoids and polyketides, immune system, and environmental adaptation (Table 3).

**Table 3:** **Statistical enrichment analysis for KEGG metabolic pathways (*p *≤0.05)**

**Pathway ID**	**Pathway**	**Genes with pathway annotation**			***p *value**
		**All genes**	**DEGs**		
			**Up**	**Down**	
ko01100	Metabolic pathways	5905 (21.49%)	607	2004	9.37E-18
ko01110	Biosynthesis of secondary metabolites	2821 (10.27%)	322	989	4.54E-16
Carbohydrate metabolism					
ko00040	Pentose and glucuronate interconversions	233 (0.85%)	31	130	3.28E-20
ko00500	Starch and sucrose metabolism	651 (2.37%)	72	257	3.70E-09
ko00053	Ascorbate and aldarate metabolism	208 (0.76%)	21	80	0.004397
ko00010	Glycolysis / Gluconeogenesis	337 (1.23%)	31	119	0.03086
ko00660	C5-Branched dibasic acid metabolism	11 (0.04%)	5	3	0.026546
ko00620	Pyruvate metabolism	247 (0.9%)	20	94	0.017667
Amino acid metabolism					
ko00360	Phenylalanine metabolism	214 (0.78%)	23	92	1.42E-05
ko00270	Cysteine and methionine metabolism	225 (0.82%)	34	78	0.000967
ko00250	Alanine, aspartate and glutamate metabolism	171 (0.62%)	21	63	0.006038
ko00300	Lysine biosynthesis	39 (0.14%)	5	21	0.000519
ko00290	Valine, leucine and isoleucine biosynthesis	90 (0.33%)	16	33	0.00268
ko00380	Tryptophan metabolism	116 (0.42%)	19	42	0.002663
ko00260	Glycine, serine and threonine metabolism	139 (0.51%)	16	52	0.013956
ko00330	Arginine and proline metabolism	167 (0.61%)	25	52	0.045321
Metabolism of other amino acids					
ko00460	Cyanoamino acid metabolism	167 (0.61%)	23	71	7.09E-06
Biosynthesis of other secondary metabolites					
ko00940	Phenylpropanoid biosynthesis	483 (1.76%)	57	201	2.29E-10
ko00941	Flavonoid biosynthesis	272 (0.99%)	29	124	1.24E-08
ko00945	Stilbenoid, diarylheptanoid and gingerol biosynthesis	241 (0.88%)	39	96	1.20E-07
ko00944	Flavone and flavonol biosynthesis	78 (0.28%)	8	37	0.000808
ko00966	Glucosinolate biosynthesis	40 (0.15%)	11	16	0.000298
ko00402	Benzoxazinoid biosynthesis	43 (0.16%)	3	24	0.001612
ko00950	Isoquinoline alkaloid biosynthesis	40 (0.15%)	5	18	0.015469
Energy metabolism					
ko00195	Photosynthesis	113 (0.41%)	4	72	1.86E-09
Lipid metabolism					
ko00592	alpha-Linolenic acid metabolism	156 (0.57%)	20	67	2.46E-05
ko01040	Biosynthesis of unsaturated fatty acids	94 (0.34%)	9	44	0.000624
ko00565	Ether lipid metabolism	675 (2.46%)	83	215	0.006119
ko00062	Fatty acid elongation	6 (0.02%)	2	4	0.003736
ko00564	Glycerophospholipid metabolism	866 (3.15%)	110	273	0.001839
ko00100	Steroid biosynthesis	80 (0.29%)	9	31	0.034756
ko00591	Linoleic acid metabolism	76 (0.28%)	5	35	0.013071
ko00071	Fatty acid metabolism	136 (0.49%)	11	56	0.012113
Metabolism of cofactors and vitamins					
ko00785	Lipoic acid metabolism	11 (0.04%)	0	9	0.005247
ko00770	Pantothenate and CoA biosynthesis	79 (0.29%)			0.045586
Metabolism of terpenoids and polyketides					
ko00904	Diterpenoid biosynthesis	98 (0.36%)	10	55	6.17E-08
ko00903	Limonene and pinene degradation	205 (0.75%)	33	83	4.46E-07
ko00906	Carotenoid biosynthesis	195 (0.71%)	25	85	1.07E-06
ko00908	Zeatin biosynthesis	312 (1.14%)	41	113	0.000209
ko00902	Monoterpenoid biosynthesis	30 (0.11%)	13	26	0.01791
Glycan Biosynthesis and Metabolism					
ko00531	Glycosaminoglycan degradation	86 (0.31%)	5	39	0.017583
Immune system					
ko04650	Natural killer cell mediated cytotoxicity	123 (0.45%)	23	42	0.00168
Signal Transduction					
ko04075	Plant hormone signal transduction	1667 (6.07%)	185	614	1.87E-13
Environmental Adaptation					
ko04626	Plant-pathogen interaction	1719 (6.26%)	139	693	3.34E-15

### Transcription factor prediction

**Figure 7:**
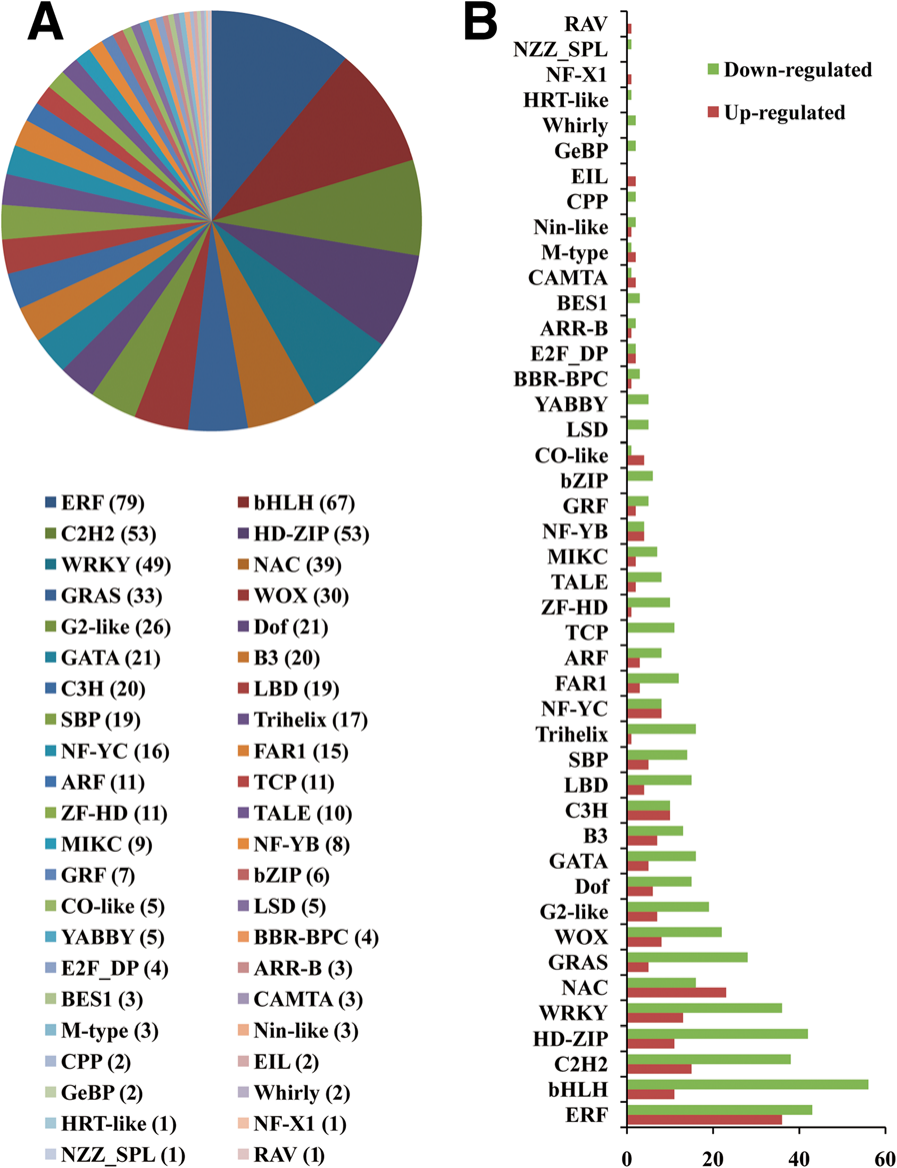
**Distribution of transcription factors in gene families. A.** The distribution of transcription factors according to the gene family information. **B.** DEGs from every gene family involved in transcription.

A total of 1636 unigenes were identified to be involved in transcription, including 720 DEGs (209 up-regulated and 511 down-regulated) (Figure 7). The largest gene family was the ethylene-responsive element binding factor family (ERF), followed by the basic helix-loop-helix family (bHLH), C2H2 family, the Homeodomain-leucine zipper family (HD-ZIP), and the WRKY family.

### Quantitative real-time reverse transcription-PCR (qRT-PCR) analysis

Our genome-wide expression analysis results were confirmed by quantitative real-time (qRT) PCR using TaqMan probes. We selected 20 unigenes, all of which are known to be related to cold stress, such as CBF (C-repeat-binding factors), LEA (Late embryogenesis abundant), LTI (low-temperature-induced), COR (cold-responsive), ERD (early dehydration-inducible), and DREB (dehydration-responsive element binding) [30,31]. The Ct values of 18S rRNA of all samples ranged from 24.0 to 26.0. All 20 transcripts showed the same expression pattern as the *in silico* differential analysis results from high-throughput sequencing (Table 4).

**Table 4:** Real-time RT-PCR with putative unique transcripts (PUTs)

**Putative unique transcript ID**	**Annotation (BLASTX)**	**Relative gene expression by qRT-PCR (2**^**-ΔΔ CT**^**)**	**Expression difference analysis of Illumina/Solexa (Log2(CT_RPKM/WT_RPKM))**
Unigene3649_All	CBF3 protein [Glycine max]	2.11 ± 0.18	7.8564
Unigene5045_All	PREDICTED: uncharacterized protein LOC100795990 isoform 1 [Glycine max]	2.64 ± 0.51	2.1173
CL9479.Contig1_All	ICE-like protein [Corylus heterophylla]	1.90 ± 0.40	2.2465
Unigene2612_All	PREDICTED: probable transcription factor PosF21-like [Glycine max]	2.70 ± 0.67	4.3792
CL26498.Contig1_All	Basic leucine zipper transcription factor [Medicago truncatula]	4.30 ± 2.79	2.0836
Unigene12211_All	PREDICTED: microtubule-associated protein 70-5-like [Glycine max]	2.82 ± 0.11	2.9144
CL25117.Contig1_All	Cold acclimation protein COR413-PM1 [Medicago truncatula]	3.51 ± 0.32	2.9919
CL33467.Contig1_All	PREDICTED: sugar transporter ERD6-like 5-like [Glycine max]	2.44 ± 0.27	1.9228
Unigene12905_All	PREDICTED: LOW QUALITY PROTEIN: sugar transporter ERD6-like 16-like [Glycine max]	12.59 ± 7.89	10.7104
CL21996.Contig1_All	PREDICTED: cysteine proteinase RD19a-like [Glycine max]	1.78 ± 0.10	1.2868
Unigene28735_All	Medicago truncatula HVA22-like protein a (MTR_4g108350) mRNA, complete cds	2.35 ± 0.28	2.107
Unigene5543_All	HVA22-like protein e [Medicago truncatula]	9.91 ± 2.45	6.7199
Unigene6814_All	PREDICTED: HVA22-like protein k-like [Glycine max]	7.02 ± 0.32	2.8637
Unigene37576_All	PREDICTED: low-temperature-induced 65 kDa protein-like [Glycine max]	2.78 ± 1.34	7.4373
CL11725.Contig1_All	PREDICTED: low-temperature-induced 65 kDa protein-like [Glycine max]	2.16 ± 0.17	7.5431
CL5093.Contig1_All	Hydrophobic protein LTI6B [Medicago truncatula]	2.12 ± 0.10	3.8777
Unigene5480_All	Hydrophobic protein RCI2A [Arabidopsis thaliana]	4.32 ± 0.32	5.8638
CL26053.Contig1_All	PREDICTED: hydrophobic protein LTI6A-like [Glycine max]	3.43 ± 0.26	1.5422
Unigene37577_All	PREDICTED: low-temperature-induced 65 kDa protein-like [Glycine max]	3.53 ± 1.07	6.5686
CL30168.Contig1_All	seed maturation protein [Glycine tomentella]	7.42 ± 0.88	3.3724

## Discussion

*Ammopiptanthus mongolicus*, the sole broad-leaved evergreen angiosperm genus in the deserts of eastern central Asia [32], can maintain its growth at temperatures as low as −30°C [15]. This attribute makes it an ideal model for studying the cold-tolerance mechanisms of plants. Several cold-related genes have been identified in *A. mongolicus*, including *AmCBL1*[7], *AmLEA*[8], and *AmCIP*[11]. However, for woody plants, the available genetic resources are not sufficient to determine the mechanisms of cold tolerance.

Few studies have performed transcriptome sequencing of *A. mongolicus*. Zhou *et al*. identified putative genes associated with drought tolerance using 454 pyrosequencing [33]. Compared with this alternative next-generation method, solexa has the advantages of lower cost and generation of larger amounts of data [17]. Moreover, using the recently developed Trinity software, any disadvantages related to short reads can be overcome, resulting in the assembly of transcriptome results without a reference genome being as reliable as those with an available reference genome [29].

During cold acclimation, plants receive low temperature signals and initiate a defense mechanism, including physical structure adaptations (changes in lipid composition), increases in intercellular osmoprotectants (such as soluble sugars, proline and betaine), and increased synthesis of anti-oxidants (superoxide dismutase, catalase and ascorbic acid reductase), enabling restoration of the balance of matter synthesis and energy metabolism and enhancing survival in colder environments [30,34]. Furthermore, expression patterns of a large number of genes during cold acclimation have been detected using gene chip and microarray technologies [35,36]. Use of transcriptome sequencing during cold acclimation will increase our understanding of the cold tolerance mechanisms of plants.

The amount of data obtained from transcriptome sequencing varies according to the transcriptome size of the species. However, the transcriptome size is affected by both gene number and abundance, and varies markedly among species. Our estimate of the nuclear genome size of *A. mongolicus* (2n = 18) using flow cytometry was 2C = 1639.12 ± 15.22 Mb. In this study, the total length of the reads from both samples was ~11.9 gigabases (Gb). This is the first report of the genome size of *Ammopiptanthus mongolicus*.

### Membrane systems

Membrane systems, which are known to be the primary site of freezing injury in plants, suffer multiple forms of damage caused by freeze-induced cellular dehydration [37]. During cold acclimation, plants experience improved cold tolerance with increased concentrations of unsaturated fatty acids and phospholipids [38]. We identified a total of 773 genes (4.46%) involved in “Lipid transport and metabolism” according to the COG classification. Moreover, according to the metabolic pathway enrichment analysis, eight pathways, including “Biosynthesis of unsaturated fatty acids” (Ko01040), were involved in lipid metabolism. Increases in the biosynthesis of unsaturated fatty acids improve cold defense and prevent damage caused by low temperatures [39]. Nine genes in this process showed significant upregulation in transcripts after cold stress. For example, the fatty acid desaturase 8 (FAD8) gene (CL23852.Contig1_All) was upregulated by 1.62-fold. The FAD8 gene in *A. thaliana* encodes chloroplast membrane-associated ω-3 desaturase, which contributes to freezing tolerance by altering the lipid composition [40].

### Intercellular osmoprotectant

Proline, one of the most important organic osmolytes, participates in the responses to various environmental stresses [41]. As a hydrophilic protein, proline can relieve the osmotic stress caused by cold-induced dehydration. Two genes (CL407.Contig3_All and CL31553.COntig1_All) encoding delta-1-pyrroline-5-carboxylate synthase (P5CS) were to be significantly upregulated by 1.42- and 3.82-fold after cold stress, respectively. As a key enzyme in proline synthesis, P5CS participates in the cold-stress response and shows high expression, which promotes the synthesis of proline for cold tolerance [42,43]. The accumulation of sucrose and other simple sugars also contributes to the stabilization of membranes, as these molecules protect membranes against freeze-induced damage *in vitro*[44]. Three LEA (late embryogenesis abundant)-related genes (CL36265.Contig1_All, CL30168.Contig1_All) were also examined with fold changes in their expression ranging from 3.37 to 5.64. The LEA protein functions as an antioxidant, as well as a membrane and protein stabilizer, during water stress [45]. In *A. mongolicus* and other legumes, recent studies have suggested that novel hydrophilic and LEA polypeptides stabilize membranes against freeze-induced injury [8,46].

### Antioxidant enzyme system

When plants are under cold stress, reactive oxygen species (ROS) accumulate. These are harmful to both the membrane and related biological macromolecules [47]. During cold acclimation, the antioxidant enzyme system of plants is enhanced in response to the increased stress [48]. *A. mongolicus* can maintain efficient growth in extremely stressful environments, which makes it a valuable natural resource and strong antioxidant [5]. The expression profiles of seven ROS-scavenging enzyme genes (*AmSOD*, *AmAPX*, *AmGPX*, *AmCAT*, *AmGLR*, *AmPrx*, and *AmTrx*) in *A. mongolicus* were reported by Shi *et al.* and *AmCAT* and *AmSOD* showed high expression levels [49]. In our study, a total of 830 DEGs were related to the GO term “oxidoreductase activity”, 186 of which were up-regulated. Among them, we identified 20 genes in the peroxisome pathway, including two (CL33833.Contig1_All and Unigene5521_All) encoding CAT (catalase) and one (Unigene12648_All) encoding SOD (superoxide dismutase) with the fold changes of 2.84, 1.42, and 4.50, respectively. In rice, CAT plays an important role in cold acclimation, and the accumulation of SODs can reduce cold injury [50].

### Ca^2+^ and ABA

As an important second messenger, Ca^2+^ is known to play a role in the plant cold-stress response. The concentration of Ca^2+^ increases rapidly during cold stress, followed by a series of signals mediated by a combinations of protein phosphorylation/dephosphorylation cascades [51]. As a large subfamily of plant kinases, Calcium dependent protein kinase (CDPKs) are implicated as important sensors of Ca^2+^ flux in plants in response to a variety of biotic and abiotic stress stimuli [52]. We have identified three genes (Unigene567_All, Unigene13378_All, Unigene13204_All) related to CDPK, with fold changes ranging from 1.96 to 3.14 in their expression after cold stress. Abscisic acid (ABA) also plays a crucial role in the cold acclimation of plants. Cold acclimation has been reported to be involved in both ABA-dependent and -independent pathways [53,54]. The type 2C protein phosphatases (PP2C) ABI1 and ABI2, which negatively regulate ABA responses, play a key role in ABA signal transduction [55]. In our study, eight DEGs (Unigene22945_All, Unigene30641_All, Unigene34701_All, CL2188.Contig1_All, CL1877.Contig1_All, CL16627.Contig1_All, CL10137.Contig1_All, and CL28715.Contig1_All) related to PP2C were identified, showing significant down-regulation with fold changes ranging from −2.3 to −12.5 in their expression after cold stress. The function of ABA-activated SnRK2 protein kinase has also been reported in dehydration stress signaling in *Arabidopsis*[56,57]. One gene (Unigene3661_All) related to SnRK2 was identified with a fold change of 2.4. On the other hand, two genes related to SnRK2 in *A. mongolicus* were reported by Zhou *et al.*, with fold changes of 1.89 and 3.75, respectively, after drought exposure for 72 h [33]. Therefore, it is likely that the SnRK2 gene of *A. mongolicus* shows an expression pattern under cold stress similar to that under drought stress. We also identified PLDα1, PLDβ1 and PLDδ to be up-regulated (fold changes of 1.20, 1.18 and 1.93, respectively). These genes are believed to function in ABA signaling in guard cells [58,59].

### Cold-related genes and transcription factors

We last focused on changes in the expression of genes associated with transcription factors. A total of 720 of 1636 unigenes were identified as DEGs, 209 of which showed significant upregulation. Most of these unigenes had homologs in other legumes, such as *Glycine max*, *Medicago truncatula* and *Lotus japonicas*, which are known to be stress-induced, such as the ERF and WRKY families. These up-regulated transcription factors may play important roles in plant defense and stress responses [60]. Some cold-related genes identified by Thomashow [31], such as COR, LEA, CBF, and DREB, have been cloned and identified by qRT-PCR showing the same trend as our Illumina/Solexa sequencing.

## Conclusions

This is the first report of transcriptome sequencing of *A. mongolicus* under cold acclimation using Illumina/Solexa. The total length of the reads was ~11.9 Gb. A total of 82795 unigenes were assembled, 32728 of which were differently expressed with 9309 unigenes showing up-regulation. By performing BLAST analysis of the all-unigenes against public databases (Nr, Swiss-Prot, KEGG and COG), we obtained functional annotations and classifications. The large number of transcriptomic sequences and their functional annotations provide sufficient resources for molecular studies of *A. mongolicus*. Moreover, information on the KEGG metabolic pathways and transcription factors will facilitate the discovery of other cold resistant genes.

## Methods

### Plant materials

*A. mongolicus* seeds were collected from AlaShan Desert in Inner Mongolia, Northwest China. After sterilization and seeding in hormone-free Murashige and Skoog medium for 30 days, the seedlings were moved and cultured at room temperature (approximately 20°C), with a photoperiod of 16 h light and 8 h dark. Two weeks after transplanting, we divided the plantlets into two groups. The first group served as the control sample (CK), while the other was moved and cultured at 4°C as the cold-treated sample (CT). Both samples were planted in the nursery of Beijing Forestry University (BJFU) (116.3°E, 40.0°N), and watered every three days. Leaves and roots of both samples were collected simultaneously after treatment for 14 days and rapidly stored at −80°C until required for RNA extraction.

### Nuclear DNA content determination

The cotyledons of *A. mongolicus* were collected from the plants from which the leaves and roots for sequencing were obtained. Cotyledons were homogenized in 2-mL homogenization buffer (45 mM MgCl_2_; 30 mM sodium; 20 mM MOPS; 0.1% (w/v) TritonX-100; pH 7.0) [61] and filtered through a 300-mesh nylon netting. The nuclei suspension was obtained by centrifuging the suspension at 1100 rpm for 6 min. After staining with propidium iodide (PI, 50 μg/mL) and incubating at 4°C for 20 min, nuclei were examined by flow cytometry. The standard sample was young healthy leaves from 1-week-old maize seedlings (*Zea mays L.*) (Zheng Dan 958). Three measurements per sample were obtained.

### RNA extraction and quality determination

Total RNA of each sample was extracted three times using a CTAB procedure [62]. Leaves and roots of each sample were extracted separately and mixed with equal amounts of mRNA after examination. The RNA samples were dissolved in 10 mM Tris (PH 7.6) and examined using the NanoDrop ND-8000; the A_260_/A_280_ ratios of both samples ranged from 1.9 to 2.1. The integrity of the RNA samples was assessed with an Agilent 2100 Bioanalyzer; no sign of degradation was found.

### cDNA library construction and sequencing

For the synthesis of cDNA and Solexa sequencing, we prepared 45 μg of total RNA for treated and control sample at concentrations of approximately 1500 ng/μl. We then enriched the poly (A) mRNA using beads with Oligo (dT) and interrupted mRNA into short fragments with fragmentation buffer. Using these short fragments as templates, we synthesized first-strand cDNA with hexamer-primers and reverse transcriptase (Invitrogen). The second-strand cDNA was synthesized using buffer, dNTPs, RNaseH (Invitrogen) and DNA polymerase I (New England BioLabs). The short fragments were then purified using a QiaQuick PCR extraction kit and resolved with EB buffer to finish the end reparation, and were connected using sequencing adaptors. After resolution by agarose gel electrophoresis, we selected fragments suitable for PCR amplification. We then constructed two paired-end libraries which were sequenced using an Illumina HiSeq™ 2000.

Raw reads produced from sequencing machines contain low-quality reads, which negatively affect subsequent bioinformatics analyses. Therefore, we discarded the these reads, including those with adaptors, those with unknown nucleotides larger than 5% and those of low quality (< 20% of the bases with a quality score Q ≤ 10) using an in-house Perl script. The average proportion of clean reads in each sample was ~91.5%, on which the following analysis was based.

### *De novo* assembly and assessment

Transcriptome *de novo* assembly was performed using the short-reads assembly program, Trinity [29], which first combined reads with certain lengths of overlap to form longer fragments, known as contigs. These reads were then mapped back to contigs. Using paired-end reads, we detected contigs from the same transcript as well as the distances between these contigs. Next, we used Trinity to connect the contigs and obtained sequences that cannot be extended on either end, known as unigenes. Finally, we used TGICL [63], a software system for rapid clustering of large EST datasets, to assemble all the unigenes from both samples to form a single set of non-redundant unigenes.

After clustering, the unigenes were divided into two classes: clusters and singletons. Finally, BLASTx alignment (E-value < 0.00001) was performed between unigenes and the protein databases, following the priority order: Nr, Swiss-Prot, KEGG and COG. The best alignment results were used to decide the sequence direction of unigenes. For unigenes that could not be aligned to any of the above databases, we used ESTScan to determine the sequence direction.

### Functional annotation

We aligned the unigene sequences to the above-mentioned protein databases by BLASTx (E-value < 0.00001) and to the nucleotide sequence database Nt (E-value < 0.00001) by BLASTn. We thus obtained proteins with the highest similarity to the given unigenes, as well as the functional annotations.

According to the Nr annotation, we obtained the GO functional annotation using the Balst2GO program [64], and the GO functional classification for all-unigenes using the WEGO software [65] to understand the distribution of gene functions of the species from the macro-level. After aligning the all-unigenes to the COG database, we obtained the COG functional annotations. Next, we further examined the complex biological behaviors. To investigate the metabolic pathway annotation of unigenes, we aligned the all-unigenes to the KEGG database [66], and so obtained pathway annotations.

### Protein-coding region prediction

To predict the protein-coding regions, unigenes were aligned to the protein databases in the above-mentioned priority order. The coding regions of proteins with the highest ranks based on the BLAST results were determined, and the coding region sequences were translated into amino acid sequences using the standard codon table. Both the nucleotide (5’-3’) and amino acid sequences of the unigene-coding region were acquired. Unigenes that could not be aligned to any of the above databases were scanned by ESTScan [67] to determine the sequence (5’-3’) direction and amino acid sequences of the predicted coding region.

### Differential expression analysis of unigenes

To identify those whose levels of expression differed, we performed a differential expression analysis of the unigenes. RPKM (reads per kb per million reads) [68] was used to calculate unigene expression levels, which eliminated the influence of gene length and sequencing level on the calculation of gene expression. The RPKM method formula was:

$$\text{RPKM}=\frac{10^{6}C}{\mathrm{NL}/10^{3}},$$

where *C* is the number of reads that uniquely aligned to one unigene; *N* is the total number of reads that uniquely aligned to all unigenes; *L* is the base number in the CDS of one unigene.

Based on the method described by Audic and Claverie [69], we determined the statistical significance of differential expression profile for each gene. FDR (False Discovery Rate) control method [70] was used in multiple hypothesis testing to correct the results for *p* value. After the FDR was obtained, we used the ratio of RPKMs to calculate the fold-change in the expression of each gene in two samples simultaneously. In our analysis, the differentially expressed genes (DEGs) were screene with the threshold of FDR ≤ 0.001 and the absolute value of log2Ratio ≥ 1 [71].

We then mapped all DEGs to each term of Gene Ontology database (http://www.geneontology.org/) and calculated the gene numbers each GO term had, and got a gene list and gene numbers for every certain GO term. Then using hypergeometric test [72], we found significantly enriched GO terms in DEGs comparing to the genome background of *A. mongolicus*. The calculated *p* value went through Bonferroni Correction, taking corrected *p* value ≤ 0.05 as a threshold. GO terms fulfilling this condition were defined as significantly enriched GO terms in DEGs. *P* value formula was:

$$P=1-\sum_{i=0}^{m-1}\frac{\left(\begin{array}{l} M\\ i \end{array}\right)\left(\begin{array}{l} N-M\\ n-i \end{array}\right)}{\left(\begin{array}{l} N\\ n \end{array}\right)},$$

where *N* is the number of all genes with GO annotation; *n* is the number of DEGs in *N*; *M* is the number of all genes that are annotated to the certain GO terms; *m* is the number of DEGs in *M*.

DEGs were also used in pathway enrichment analysis. We calculated the gene numbers in each pathway by mapping all DEGs to KEGG database (http://www.genome.jp/kegg). By comparing with the whole genome background of *A. mogolicus*, pathways with *p* ≤ 0.05 were chosen as significantly enriched in DEGs, using the same multiple testing correction method with GO enrichment analysis. With pathway enrichment analysis, we got the main biochemical pathways and signal transduction pathways in which DEGs involved. Some cold stress related pathways were listed, on which all following analyses and discussions based.

### Transcription factors analysis

Transcription factors were predicted according to protein sequences obtained from CDS prediction. We used hmmsearch to search the domain of the plant transcription factors (http://plntfdb.bio.uni-potsdam.de/v3.0/) and classified unigenes according to the gene family information. Genes that were believed to be associated with cold stress were selected for further investigation.

### Quantitative real-time PCR analysis

The unigenes selected were then assessed by quantitative real-time PCR. Approximately 1 μg of total RNA of each sample was converted into single-stranded cDNA using M-MLV Reverse Transcriptase (Progema, USA). The cDNA products were then diluted 100-fold with deionized water before use as a template. The reaction was performed on a STEP ONE PLUS™ Real-Time PCR System (Applied Biosystems, USA) using the RealMasterMix (SYBR Green) (China). The reaction system (20 μL) contained 9-μL 2.5 × RealMasterMix/20 × SYBR Solution, 1 μL of each of the forward and reverse primers and 1-μL cDNA template. The reaction was performed under the following conditions: 94°C for 2 min, followed by 45 cycles of 94°C for 20 s, 60°C for 35 s and 68°C for 1 min. Three independent biological replicates were performed for each sample. Expression levels of the selected unigenes were normalized to that of 18S rRNA, an internal reference gene. Relative gene expression levels were calculated using the 2^-ΔΔCt^[73]. The primer sequences used for qRT-PCR are listed in Additional file 4.

## Competing interest

The authors declare that they have no competing interests.

## Authors’ contributions

WLY and XLX conceived this study. TP designed the experimental plan. TP and CYY participated in sample collection, RNA preparation and analyzed the sequence data. All authors read and approved the final manuscript.

## Supplementary Material

Additional file 1 **100 most abundant transcripts in both samples.** The list of 100 most abundant transcripts in both cold-treated sample (CT) and control sample (CK). The Nr annotations of each gene-ID were also listed. Click here for file

Additional file 2 **Genes that expressed only under cold treated.** The list of genes that expressed only under cold treated. The gene length, raw reads, as well as functional annotation of several public databases were also listed. Click here for file

Additional file 3 **GO enrichment analysis.** The list of the GO enrichment analysis results. GO terms with a corrected *p* value ≤ 0.05 were defined as significantly enriched in DEGs. Click here for file

Additional file 4 **Primer sequences for qRT-PCR.** The primers used in quantitative real-time PCR analysis. Click here for file
