# Supplementary material for: De novo sequencing and transcriptome analysis of the desert shrub, Ammopiptanthus mongolicus, during cold acclimation using Illumina/Solexa
Source: BMC Genomics. 2013 Jul 18;14:488. doi: 10.1186/1471-2164-14-488 (PMC3728141; doi:10.1186/1471-2164-14-488)
Supplement: Additional file 4 — Primer sequences for qRT-PCR. The primers used in quantitative real-time PCR analysis. [file 1471-2164-14-488-S4.docx]

**Additional file 4. Primer sequences used for qRT-PCR.**

| **Gene** | **Forward primers (5’ to 3’)** | **Reverse primers (5’ to 3’)** |
| --- | --- | --- |
| Unigene3649_All | ACTGGGATTTCAGCATATTCAC | GTTGTTAGGGACTCTCATCTCG |
| Unigene5045_All | GTGATTCTGATTCTTGTGCTGA | CCGTATTATGTGATCGTCTGTG |
| CL9479.Contig1_All | CAAATCCACCACTTTGTCTTCT | TGCTTGTAGCTGCTAACTGTGT |
| Unigene2612_All | GCAAATAACTAAGGTGCCAAAG | CCTCCATGTCCCAGAGATAATA |
| CL26498.Contig1_All | TGCTAGACTCAAAAGACAGCAA | GATTCAAGCGTGGAAGAATTAC |
| Unigene12211_All | TTCTATGGCCTTATCCTTCAGA | CAACATATTGATGCACTGCTCT |
| CL25117.Contig1_All | ATAACAGCAACAGAAGCAATCC | TCAACTCTGATCTCAGGGAACT |
| CL33467.Contig1_All | TTGTTATACCGAAGGACTTGCT | AGACCAAGGGACGTACACTAGA |
| Unigene12905_All | CCTTGGATTCTCCACTTTTAAC | TTGTACTTGTACCATGCCAGAC |
| CL21996.Contig1_All | AGGGAAACCACACTGAAATTAG | TTACCAGTGTGATCCAGAAGAA |
| Unigene28735_All | AATTTCTTGCCCACTATACGTC | GTGAACTGCTTCCATCTTTAGC |
| Unigene5543_All | CAATAGCTTCACATCGTACCAA | GTCACTTTCCTCTTCCAATCAG |
| Unigene6814_All | GCAGATCAAACTTGTCAGTTCA | GGTTGGCAGTAAGTCAAGTCTC |
| Unigene37576_All | AACTGGTACCAATACTGCTGCT | CATCCTCTTTTTCCACTGTCTC |
| CL11725.Contig1_All | TTCTCTACCACCTTTCTCTCCA | GAAAATTCAGAACCAGCTGAAC |
| CL5093.Contig1_All | GCCCTTGACAACTGTTTATTGT | GACCACAGCTACCTTCGTAGAC |
| Unigene5480_All | GACCACAGCTACCTTCGTAGAC | GCCCTTGACAACTGTTTATTGT |
| CL26053.Contig1_All | AGACATCCTCCTTGCTATCATC | GCAGAGTTGAAAGACATGAACA |
| Unigene37577_All | ATGAATCAGTGGAGAAACCATC | TCCCATAAACTGGAGAAGCTAA |
| CL30168.Contig1_All | GTTTGACCAGATGTCAACAATG | AGTGTTCGTTCCAGTAGGATGT |
| 18S | GGCTCTGCCCGTTGCTCT | CGTCACCCGTCACCACCA |
